# A machine learning screening model for identifying the risk of high-frequency hearing impairment in a general population

**DOI:** 10.1186/s12889-024-18636-1

**Published:** 2024-04-25

**Authors:** Yi Wang, Xinmeng Yao, Dahui Wang, Chengyin Ye, Liangwen Xu

**Affiliations:** 1 Department of Epidemiology and Biostatistics, School of Public Health, Hangzhou Normal University, Hangzhou, 311121 Zhejiang China; 2 Hangzhou Center for Disease Control and Prevention, Hangzhou, Zhejiang China; 3 Department of Health Management, School of Public Health, Hangzhou Normal University, Hangzhou, Zhejiang China

**Keywords:** Hearing impairment, Machine learning, General population, Questionnaire-based indicators, Blood parameters

## Abstract

**Background:**

Hearing impairment (HI) has become a major public health issue in China. Currently, due to the limitations of primary health care, the gold standard for HI diagnosis (pure-tone hearing test) is not suitable for large-scale use in community settings. Therefore, the purpose of this study was to develop a cost-effective HI screening model for the general population using machine learning (ML) methods and data gathered from community-based scenarios, aiming to help improve the hearing-related health outcomes of community residents.

**Methods:**

This study recruited 3371 community residents from 7 health centres in Zhejiang, China. Sixty-eight indicators derived from questionnaire surveys and routine haematological tests were delivered and used for modelling. Seven commonly used ML models (the naive Bayes (NB), K-nearest neighbours (KNN), support vector machine (SVM), random forest (RF), eXtreme Gradient Boosting (XGBoost), boosting, and least absolute shrinkage and selection operator (LASSO regression)) were adopted and compared to develop the final high-frequency hearing impairment (HFHI) screening model for community residents. The model was constructed with a nomogram to obtain the risk score of the probability of individuals suffering from HFHI. According to the risk score, the population was divided into three risk stratifications (low, medium and high) and the risk factor characteristics of each dimension under different risk stratifications were identified.

**Results:**

Among all the algorithms used, the LASSO-based model achieved the best performance on the validation set by attaining an area under the curve (AUC) of 0.868 (95% confidence interval (CI): 0.847–0.889) and reaching precision, specificity and F-score values all greater than 80%. Five demographic indicators, 7 disease-related features, 5 behavioural factors, 2 environmental exposures, 2 hearing cognitive factors, and 13 blood test indicators were identified in the final screening model. A total of 91.42% (1235/1129) of the subjects in the high-risk group were confirmed to have HI by audiometry, which was 3.99 times greater than that in the low-risk group (22.91%, 301/1314). The high-risk population was mainly characterized as older, low-income and low-educated males, especially those with multiple chronic conditions, noise exposure, poor lifestyle, abnormal blood indices (e.g., red cell distribution width (RDW) and platelet distribution width (PDW)) and liver function indicators (e.g., triglyceride (TG), indirect bilirubin (IBIL), aspartate aminotransferase (AST) and low-density lipoprotein (LDL)). An HFHI nomogram was further generated to improve the operability of the screening model for community applications.

**Conclusions:**

The HFHI risk screening model developed based on ML algorithms can more accurately identify residents with HFHI by categorizing them into the high-risk groups, which can further help to identify modifiable and immutable risk factors for residents at high risk of HI and promote their personalized HI prevention or intervention.

**Supplementary Information:**

The online version contains supplementary material available at 10.1186/s12889-024-18636-1.

## Introduction

Hearing impairment (HI) is the most common sensory impairment and has become a public health concern worldwide [1, 2]. HI is often considered an invisible disability. It is characterized by reduced hearing sensitivity and loss of speech understanding caused by degeneration of the cochlea, the auditory nerves or both [3]. This disorder has caused substantial social and economic burdens on adults in China. In 2011, 2013, and 2015, the direct costs attributable to HI of middle-aged and older people aged 45 and above in China were $50.699 billion, $81.783 billion and $106.777 billion, accounting for 3.43, 4.54 and 5.54% of the overall healthcare costs in the same year, respectively [4]. Currently, pure-tone hearing tests are the gold standard for identifying HI, which requires expensive audiological equipment and trained professionals, leading to limitation the feasibility of utilizing pure-tone hearing tests for mass screening of HI at the community level. Moreover, relevant studies have shown that due to the lack of professional hearing health care personnel and insufficient allocation of HI detection equipment in China's grassroots communities, residents generally lack routine HI detection services, resulting in timely detection of early HI lesions and high-risk group [5, 6]. Therefore, there exists a pressing need to establish a practical and accessible screening tool tailored for residents with HI, with the aim of mitigating the progression to clinically significant HI [7]. Many HIs, defined as audiometric losses, usually start from higher-frequency deterioration, then develop slowly and evolve gradually to lower-frequency or speech-frequency dysfunction [8]. Primary screening for high-frequency hearing impairment (HFHI) could be the key to starting early prevention and intervention.

Currently, well-known HI risk factors in the general population include age [9], genetics, behavioural factors (e.g., smoking and exercise) [10], environmental exposures (e.g., noise exposure), health care utilization factors (e.g., immunization and antibiotics), and chronic disorders (e.g., hypertension and diabetes) [11]. Furthermore, various biomarkers involved in inflammation (such as increased white blood cell (WBC) counts, neutrophil (NE) counts, monocyte (MO) counts, and lymphocyte (LY) counts) and metabolic parameters (such as low-density lipoprotein (LDL) and high-density lipoprotein (HDL)) are also recognized as risk indicators of HI [12]. Chronic changes in inflammatory status that accompany the ageing process, known as inflammation, may cause or accelerate long-term damage to the hearing system [13]. Red blood cell distribution width (RDW), a parameter used to classify anaemia, was recently reported to be associated with inflammation and microcirculation disorders [14]. HDL and LDL have been reported to affect blood supply and thus may potentially influence sudden sensorineural HI [12]. While several studies have attempted to investigate the relationships between HI and blood inflammation and metabolic parameters, limited studies have used these parameters to predict HI. Notably, in recent years, Chinese community residents have regular annual physical examinations, and blood tests (blood routine and biochemical) during physical examinations are routine tests, and doctors usually extract only a small part of the information hidden in the results of routine blood tests [15]. Thus, in the era of big data, conducting more comprehensive analyses by integrating existing community blood test data with questionnaire data holds significant promise for elucidating HI-related biomarkers, identifying individuals with HI, and proactively alerting community residents about their risk of HI.

In the area of HI assessment, various tools, such as screening scales, apps, tablet-based and computer-based devices, and internet-based platforms, have been developed by researchers. While these products are widely utilized in patients with HI, the existing assessment scales primarily focus on evaluating the functional aspects and consequences of HI, especially in the elderly population [16]. Despite the availability of low-cost automated hearing tests for remote screening, their accuracy and accessibility are constrained by factors such as environmental noise, equipment reliability, and data security [17].

Machine learning (ML), situated at the intersection of statistics and computer science, is a scientific field dedicated to understanding how computers learn from data. ML algorithms, which are capable of capturing complex and unpredictable patterns in human physiology, have shown promise in audiology [18]. To date, most HI prediction models based on ML algorithms have been built for noise-related workers, and their predictors are usually noise exposure level and exposure time, which are not suitable for the general population [19]. The existing HI prediction models for the general population are limited. One study established a simple screening tool for moderate-to-severe HI in Shanghai communities, and the accuracy of the testing set reached 91.92%, but the study subjects were mainly elderly people [20]. Another study predicted HI based on a decision tree-based algorithm and obtained an area under the curve (AUC) value of 0.870, but this study mainly focused on speech-frequency HI and limited the early prevention of HI [21].

Therefore, this study aimed to develop an HFHI ML risk screening model for the general population by using routine physical examination data (blood biomarkers and liver function indicators) and demographic and lifestyle risk factor data from community residents. The performances of the seven most commonly used ML algorithms for screening HFHI in the general population were further compared to determine the best algorithm. We envision that this model can be used directly in community settings to screen populations for HFHI risk in combination with electronic case information to provide guidance and assistance for personalized HFHI prevention or intervention in the community.

## Materials and methods

### Study design and dataset

**Fig. 1:**
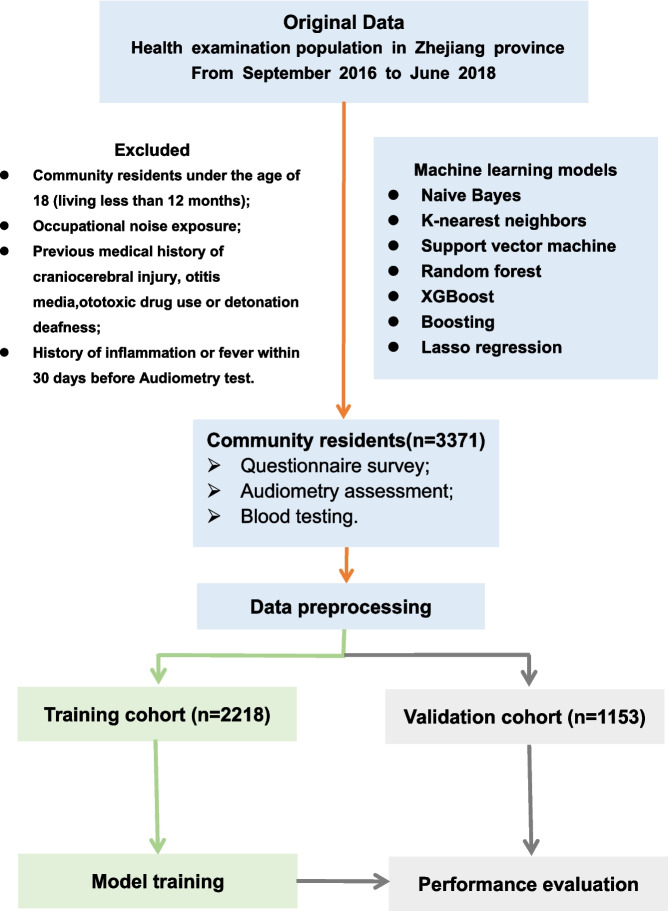
Flow diagram of study. Model development performed with 3371 community residents

To develop the optimal HI screening model, a multistage stratified cluster sampling method was used to conduct a population-based cross-sectional survey of 7 health centres in Hangzhou, Jiaxing, Huzhou, Quzhou and Lishui from 2016 to 2018 based on the geographical distribution of Zhejiang Province and the stratification of city size, city and county. Considering the sample effective rate and the prevalence rate of HFHI, the sample size was calculated to be at least 1436 people by using the current study formula, and 4010 people were ultimately investigated. After deleting and filling in the missing data, 3371 people were included. During the specified timeframe, all individuals undergoing physical examinations at these health centres were asked to ascertain their willingness to participate in our study. Those who agreed to participate were included in the study and asked to sign an informed consent form. After completing the informed consent process, all participants completed a comprehensive assessment, including a questionnaire survey, an audiometric assessment and a blood test, during their annual physical examination (Fig. 1). Inclusion criteria are as follows: (1) Residents from Zhejiang Province who have lived in the area for more than one year and are over 18 years old; (2) No self-reported occupational noise exposure; (3) No previous medical history of otitis media, craniocerebral injury, or ototoxic drug use or detonation deafness; (4) No history of inflammation or fever within 30 days before audiometry test. The study was conducted according to the guidelines of the Declaration of Helsinki, and approved by the Institutional Review Board of Hangzhou Normal University Ethics Committee (grant number: 2017LL107), and all personal privacy information was well protected and removed during the process of analysis and publication.

### Audiometric assessment

All pure-tone air-conduction hearing thresholds were measured by trained researchers using audiometers (AT235; Interacoustics AS, Assens, Denmark) with supra-aural headphones (TDH-39; Telephonic Corporation, Farmingdale, USA). Subjects were advised to stay away from noisy conditions for more than 12 h prior to the hearing test to improve test accuracy. Pure-tone air conduction hearing thresholds were tested in both ears at frequencies of 3 Kilohertz (kHz), 4 kHz, 6 kHz and 8 kHz over an intensity range of -10 to 110 decibel (dB(A)). In the audiometric examination, participants who did not respond at least once were considered nonresponsive. To measure the reliability of participants' responses, the 1 kHz frequency was tested twice in each ear. An unreliable response was considered if the results differed by more than 10 dB(A), and then the assessment was performed again. Pure tone averages were computed across fixed frequency bands (3 kHz, 4 kHz, 6 kHz and 8 kHz) or across affected frequencies, and the result of > 25 dB(A) with a worse-hearing ear was diagnosed as HFHI [22].

### Questionnaire survey

The questionnaire survey and audiometry test were conducted on the same day. The original questionnaire was developed and revised through expert consultation and included questions about demographics, symptom and disease histories, behavioural factors, environmental exposure and cognitive parameters (Additional file 1). Then, a pre-participation survey involving 926 participants was administered. The results showed that the developed questionnaire reached a Cronbach's α coefficient of 0.753 and a Kaiser–Meyer–Olkin (KMO) value of 0.794 [23–25].

### Blood sample collection and laboratory testing methods

First, research participants adhered to fasting conditions before morning blood collection, during which medical personnel obtained peripheral venous blood samples (the same day as the audiometric assessment and questionnaire survey). Each sample received a unique serial number corresponding to the participant's questionnaire. On-site investigators utilized sterile syringes to carefully extract 1 ml of daily collected EDTA-anticoagulated whole blood. The collected blood was subsequently distributed into sterile Eppendorf tubes, sealed, and subjected to a meticulous verification process involving cross-checking the participant's name and serial number. Finally, each appropriately labelled sample was securely stored at -80 °C for subsequent analysis.

### Candidate indicators

To develop HFHI risk screening models, a total of 68 variables were collected from the questionnaire survey and blood test for modelling purposes. Demographics, symptom and disease histories, behavioural factors, environmental exposure, and hearing cognition were included in the structured questionnaire. The data were collected and measured under standardized conditions following uniform procedures. The details of the candidate indicators are shown in Table 1.

**Table 1:** Variables included in this study

**Data Sources**	**Category**	**Number of indicators**	**Candidate indicator (s)**
Questionnaire-based indicators	Demographics	6	age, gender, marital status, education level, personal average monthly income, familial disease
	Symptom histories	3	self-perceived hearing status, tinnitus, ear pain history in the past year
	Disease histories	11	hypertension, diabetes, cerebral haemorrhage, arteriosclerosis, cerebral infection, anemia, migraine, coronary heart disease, otitis media, chronic kidney disease, tumors
	Behavioral factors	7	smoking, secondhand smoking, alcohol drinking, hours of sleep, electronic volume, daily fruit and vegetable intaking, exercise frequency
	Environmental exposure	4	workplace noise exposure, living noise exposure, work stress, life stress
	Hearing cognitive situation	4	pay attention to your hearing, pay attention to hearing protection, regular hearing check, hearing protection skills
Blood parameters	Blood routine indices	21	eosinophil(EO), basophilic(BA), EO(%), hemoglobin(HGB), lymphocyte(LY), mean corpuscular hemoglobin concentration(MCHC), monocyte(MO), mean platelet volume(MPV), neutrophil(NE), blood platelet count(BPC), RDW, basophilic(%)((BASO(%)), hematocrit(HCT), LY(%), mean corpuscular hemoglobin(MCH), mean corpuscular volume(MCV), MO(%), NE (%), platelet distribution width(PDW), red blood cell(RBC), WBC
	Hepatic function indices	12	triglyceride(TG), alanine aminotransferase(ALT), indirect bilirubin (IBIL), direct bilirubin(DBIL), albumin(ALB), total bilirubin(TBIL), blood urea nitrogen(BUN), aspartate aminotransferase(AST), total cholesterol(TC), LDL, HDL, creatinine(CR)

### Statistical analyses

#### Data pre-processing and feature selection

Epidata V.3.1 was used for survey data entry, checking and error correction. All the statistical analyses were carried out using R software V.4.3.2. For data pre-processing, we first excluded participants for whom more than 5% of the haematological data were missing. Second, for participants who had less than 5% missing data for haematology features, we imputed the missing data within each health centre using values generated by multiple interpolation without changing the distribution of the observed data [26]. Overall, 3371 participants were included in the cohort. Third, since the normal reference value ranges varied in haematology tests of different health centres (Additional file 2), for the purpose of future generalizability of the screening model, we recoded the haematological test indicators into three-level categorical values (i.e., low, normal and high) according to their corresponding reference values. Finally, a total of 3371 subjects and 68 covariates were included in the subsequent analysis. Univariate comparisons were conducted between HFHI patients and controls. Categorical variables are shown as n (%). Differences among groups at baseline were analysed by the chi-square test (for categorical variables). All *p* values were two-tailed, and *p* < 0.05 was considered to indicate statistical significance at this stage.

#### Model development and validation

After performing univariant feature selection, 58 indicators were selected for subsequent model construction. To both construct and evaluate the models, 2/3 (*n* = 2218) of the study population was randomly generated as the training set, and the remaining 1/3 (*n* = 1153) was used as the validation set (Fig. 1). Seven different ML algorithms were adopted in the training set to construct 7 distinct predictive models. These algorithms include naive Bayes (NB) [27], K-nearest neighbours (KNN) [28], support vector machine (SVM) [29], random forest (RF) [30], eXtreme Gradient Boosting (XGBoost) [31], Boosting [32], least absolute shrinkage and selection operator (LASSO regression) [33] (packages “randomForest”, “e1071”, “FNN”, “gbm”, “xgboost”, “caret”, and “glmnet” in R statistical analysis software). In addition, to evaluate the validity of each model, we used fivefold and tenfold cross-validation techniques on the entire dataset. Specifically, for fivefold cross-validation, the available dataset was divided into five roughly equal-sized subsets. Four of them were applied to fit the model, and the remaining one was used to estimate the accuracy of the model. Similarly, for the tenfold cross-validation, we performed 10 cross-validations by randomly dividing the entire dataset into 10 parts for 10 iterations. In each iteration, we selected 9 parts as training data and 1 part as the test set. The average result was 10% of the test data unused for each model.

The evaluation of the model was completed in the validation set. For two class-predictions these are typically the true positives (TP), true negatives (TN), false positives (FP) and false negatives (FN). The performance of the developed models was assessed by six metrics: AUC, accuracy ($\frac{TP+TN}{TP+TN+FP+FN}$), precision ($\frac{TP}{TP+FP}$), recall ($\frac{TP}{TP+FN}$), specificity ($\frac{TN}{TN+FP}$), and F-score ($\frac{2TP}{2TP+FP+FN}$). The AUC value was used as a reasonable summary of the overall diagnostic accuracy of the test, and the model with the largest AUC value was considered to have the best overall performance. Accuracy was defined as the percentage of true outcomes out of all prediction results. Precision was the ratio of true positives among all positive results [34]. Recall was the ratio of true positives out of true cases. The specificity reflects the percentage of true negatives among all negative results identified by the HFHI screening model. The F-score was the harmonic average of the precision and recall, allowing for comparison of the models' performance in identifying true positives when cases and controls were unbalanced in the dataset. By convention, the threshold for the AUC of a good model was set to 0.70 [35]. After that, the model with the best performance in terms of the six accuracy measurements was selected as the final HFHI screening model.

Finally, based on the variables that survived in the final model, we used the "rms" and "nomogram" R packages to construct a nomogram. The nomogram was based on proportionally converting each regression coefficient in multivariate logistic regression to a 0- to 100-point scale [36]. The effect of the variable with the highest *β* coefficient (absolute value) was set to 100 points. The points were added across variables to derive total points and then converted to the nomogram score, representing a certain individual's model-based probability of having HFHI.

## Results

### Subject characteristics and prevalence of HFHI

The present study included 3371 community participants, which consisted of 1730 males (51.3%) and 1641 females (48.7%), aged between 18 and 98 years, with a mean of 50.39 ± 15.23 years. Among them, 57.3% (1930/3371) were diagnosed with HFHI. Compared to those without HFHI, those with HFHI were usually older; more likely to be male; had lower education levels and incomes; and were more likely to be diagnosed with hypertension, diabetes, otitis media and chronic heart disease. The univariate analysis results of the behavioural factors, environmental exposures, symptoms and disease conditions, routine blood indices and hepatic function indices are summarized in Additional file 3. As a result, a total of 58 candidate indicators were identified for the next process of model construction (*p* < 0.05).

### Performance evaluation of the HFHI screening models

**Fig. 2:**
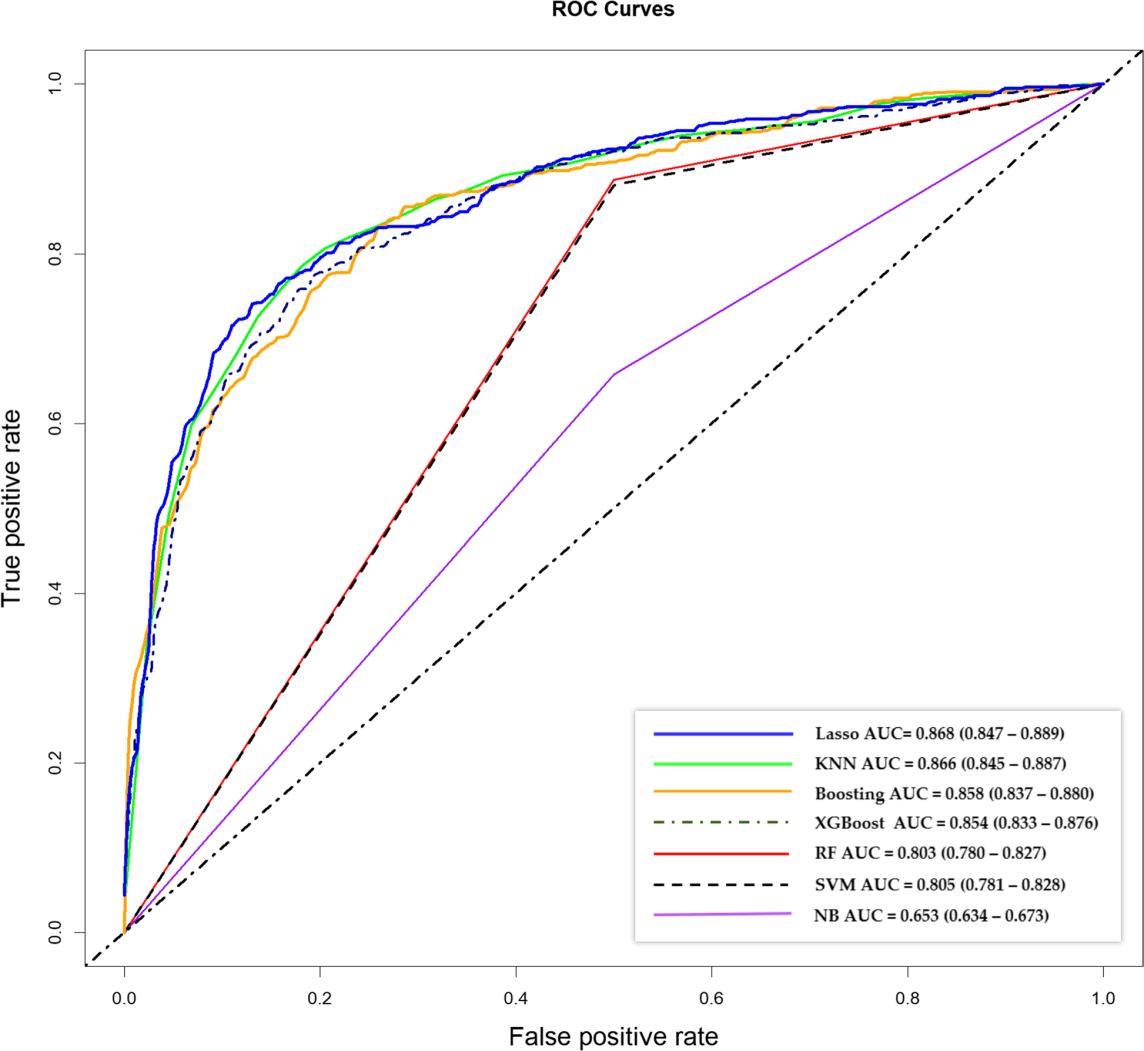
HFHI predictive performance of seven machine learning classification models

In this study, we used 7 ML algorithms to construct the HFHI classification models in the training set and evaluated the performance of these models in the validation set using AUC values, accuracy, precision, recall, specificity and F-score measurements. Finally, the model with the best discriminative ability in the validation stage was selected as the final model to distinguish HFHI patients from community residents. We first compared the performance of each model by using commonly used receiver operating characteristic (ROC) curves and AUC values. The validated ROC curves and the validated AUC values for all models are shown in Fig. 2 and Table 2. Among the adopted algorithms, the LASSO algorithm achieved the best validated AUC of 0.868 (95% confidence interval (CI): 0.847–0.889) in the validation cohort, indicating that it had the best overall discriminative ability compared to the other models. In addition, KNN, Boosting and XGBoost achieved relatively good overall discriminative ability, with validated AUC values of 0.866 (95% CI: 0.845–0.887), 0.858 (95% CI: 0.837–0.880) and 0.854 (95% CI: 0.833–0.876), respectively.

**Table 2:** Comparisons on accuracy, precision, recall, specificity and F-score of classification among different machine learning approaches

**Performance metric**	**NB**	**SVM**	**RF**	**XGBoost**	**Boosting**	**KNN**	**LASSO**
ROC-AUC	0.653	0.805	0.803	0.854	0.858	0.866	0.868
Accuracy, %	59.76	80.49	80.57	77.02	77.97	79.10	79.44
Precision, %	**93.52**	**85.47**	84.73	80.80	84.00	83.28	**85.53**
Recall, %	34.02	80.56	81.74	79.97	77.32	80.71	78.35
Specificity, %	**96.62**	80.38	78.90	72.78	78.90	76.79	81.01
F-score, %	49.89	82.94	83.21	80.38	80.52	81.97	81.78

Values greater than 85% are highlighted in bold

These ML models were further evaluated and compared with respect to other performance-related properties, including accuracy, precision, recall, specificity and F-score. The results are summarized in Table 2. In detail, the best accuracy was attained by the RF model (80.57%). In terms of precision, all 7 models achieved relatively good performance by attaining a precision of more than 80%, of which the best model was the NB model (93.52%). For the measurement of recall, the RF models attained the best value of 81.74% among the 7 models. The two best models in terms of specificity were the NB model (96.62%) and the LASSO model (81.01%). Finally, we compared the F-scores among these models, where 6 out of 7 models achieved values above 80%. The RF model (83.21%), the SVM model (82.94%), the KNN model (81.97%), the LASSO model (81.78%), the Boosting model (80.52%), and the XGBoost model (80.38%) are ranked from highest to lowest.

After performing these comprehensive comparisons, we found that the overall performances of the SVM model, RF model, KNN model and LASSO regression model were relatively better than those of the other models, but the AUC values of the SVM model (0.805), RF model (0.803) and KNN model (0.866) were lower than those of the LASSO regression model. Furthermore, we used fivefold and tenfold internal cross-validation methods to evaluate the performance of different algorithms. The AUC values of the models from cross-validation were compared with those of the original model. Similar to the original model, LASSO and KNN consistently outperformed the other algorithms. Specifically, in the fivefold cross-validation, the mean AUC values of the LASSO- and KNN-based models were both 0.857. Notably, when comparing the 95% CI, the KNN model showed a slightly narrower range (0.844–0.869) than did the LASSO model (0.845–0.870). In the tenfold cross-validation model, KNN achieved a slightly better performance than LASSO in terms of the AUC mean (Additional file 4). However, from the perspective of model interpretation and application, the LASSO-based model has unique strengths. First, in terms of model application, LASSO is capable of selecting impactful variables, thereby simplifying the model and facilitating its application value. KNN lacks a variable filtration step, necessitating the inclusion of all variables for subsequent application. Second, in terms of model interpretation, the important variables selected by LASSO can be used to design personalized intervention programs, while the KNN-based model does not have such capability since all involved features contribute to the model without a clear distinction of importance, limiting interpretability and customization potential. Consequently, we finally adopted the LASSO regression algorithm to construct the final HFHI screening model.

By adopting LASSO regression, our screening model ultimately selected 34 variables as indicators of HFHI risk and reached a fitted AUC of 0.866 (95% CI: 0.852–0.881) in the training cohort and a predicted AUC of 0.868 (95% CI: 0.847–0.889) in the validation cohort. The two AUC values were relatively high and differed very little, indicating that the derived model attained robust performance. The 34 HFHI risk indicators that survived in the final LASSO-based model included 5 demographic indicators, 7 disease-related features, 5 behavioural factors, 2 environmental exposures, 2 hearing cognitive factors, and 13 blood test indicators. Among them, history of coronary heart disease, otitis media and self-reported hearing issues, as well as several routine blood indices (e.g., RDW, PDW and LY%) and liver function indicators (e.g., TG, IBIL, AST and LDL), were identified as the most significant indicators (Additional file 5).

### The HFHI screening nomogram and a case study for model interpretation

**Fig. 3:**
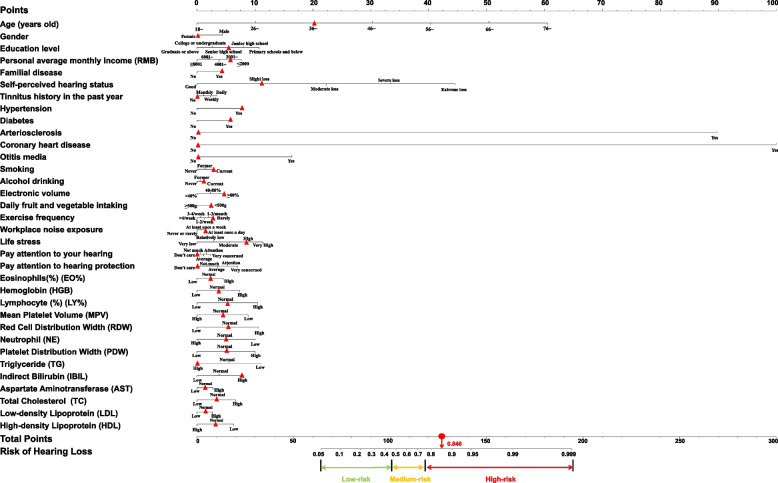
Nomogram for predicting the probability of HFHI in community setting. The first row (Points) indicates the points that are assigned to each variable's measurement from rows 2–35, which are the variables that are included in the risk model. The assigned points for all variables are then summed, and the total points is shown as the total score. Once the total score is located, draw a vertical line down to the bottom line to obtain the predicted probability of HFHI. The patient record shows a male with HFHI and the red triangle is the characteristics of this case and its corresponding predicted risk score

Based on the 34 HFHI risk indicators identified by LASSO regression, we further generated a nomogram to transform the LASSO regression model into an accessible screening tool that could ultimately be easily used by primary care physicians and community residents. In the nomogram (Fig. 3), an individual's total points were obtained by summing the points of each individual indicator achieved by a certain individual on the corresponding scale, and a vertical line was drawn on the scale based on the total points, providing this individual's final HFHI risk score. In our dataset, all individuals were ranked from low to high risk based on their risk scores and classified into three different risk groups: high-risk (score 0.75–1.00), medium-risk (score 0.45–0.75), and low-risk (score 0–0.45). The classification threshold of each risk category was determined based on the positive predictive value (PPV), sensitivity and specificity of each risk category. In general, the low-risk group comprised 1314 individuals, 22.91% (301/1314) of whom exhibited HFHI. In the medium-risk group, consisting of 822 individuals, 60.83% (500/822) were diagnosed with HFHI. Within the high-risk group, 91.42% (1129/1235) were confirmed as having HFHI. According to the nomogram, the demographic characteristics of the high-risk groups of HFHI patients could be described as elderly, low-educated, and low-income males. In terms of disease history, people with a history of tinnitus, hypertension, diabetes, coronary heart disease, or otitis media were at greater risk of HFHI. For lifestyle features, a history of smoking, alcohol consumption, high-level volume when using electronic products, high-level life pressure, and noise exposure in the working environment were risk factors for HFHI. Furthermore, 13 blood test indicators were identified by our model. Among them, the indicators that had the greatest impact on the HFHI were RDW, NE and TG.

In Fig. 3, we also use a community resident as an example to illustrate the application of the constructed HFHI screening model in community settings, showing the model's ability to identify potential high-risk residents who are normally overlooked. Generally, senior residents were at greater risk of HFHI, as age was one of the most important indicators of HI. However, for the 42-year-old man in our validation set, he was relatively young but was classified into the high-risk group of HFHI, as his identified HFHI risk was 0.846. Moreover, this middle-aged man was confirmed to have HFHI according to his audiology test results (47.50 dB for the poor ear). As shown in Fig. 3, the 34 involved features are marked with red triangles based on the patient records. From the perspectives of demographic and disease status, this man had self-perceived HI, hypertension and diabetes. He also has several behavioural risk factors, including smoking, alcohol consumption, excessive electronic volumes and daily noise exposure in the workplace. In terms of biomarker risk factors, the patient had an abnormally high level of IBIL. It is hoped that by applying this screening model, we could screen these potentially high-risk patients for subsequent confirmatory diagnosis and identify their risk factors for subsequent individualized interventions.

### Characteristics differences in risk stratification

#### Differences in resident lifestyles according to risk stratification

**Fig. 4:**
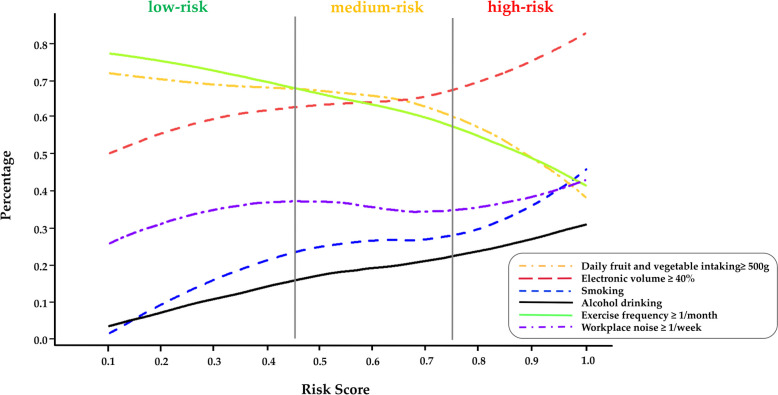
Distribution of resident lifestyle in risk stratification

To further explore the distribution of the lifestyle variables identified by the model in the three risk categories, we calculated the proportion of individuals with certain lifestyle behaviours within intervals of 0.1 points for their risk score and plotted loess curves over the spectrum of risk profiles. As shown in Fig. 4, the proportion of individuals with habits of using electronic volume over 40%, smoking, alcohol consumption, or self-reported workplace noise experience more than once a week increased significantly with increasing risk scores, while the proportion of individuals with habits of daily fruit and vegetable intake over 500 g and exercise more than once per month decreased significantly. In detail, 72.83% (957/1314) of individuals in the low-risk group exercised more than once per month, while this proportion decreased to 46.07% (569/1235) in the high-risk group. A total of 28.10% (347/1235) of individuals in the high-risk group drank alcohol, which was 3.13 times greater than that of the low-risk group (8.98%, 118/1314). Similarly, 39.51% (488/1235) of individuals in the high-risk group were smokers, which was 2.93 times greater than that of the low-risk group (13.47%, 177/1314).

#### Differences in the resident blood indices according to risk stratification

**Fig. 5:**
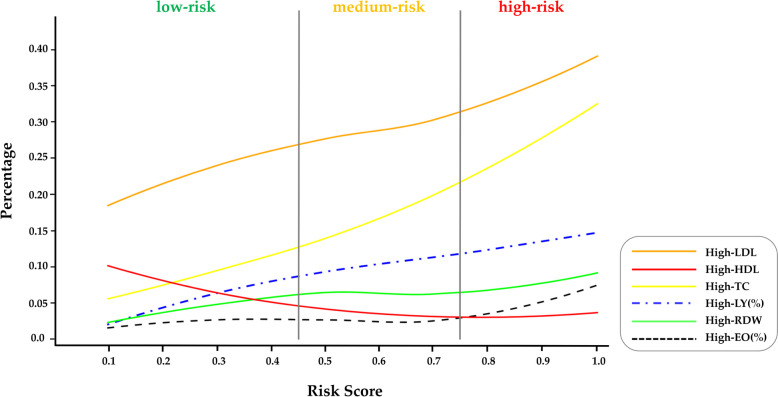
Distribution of resident blood index in risk stratification

We also explored the distribution of the identified blood index variables in the three risk categories (Fig. 5). As the risk score increased, the proportion of individuals with high levels of LDL, LY%, RDW, TC and EO% also increased, while the proportion of individuals with high levels of HDL decreased slightly. Among them, 29.15% (360/1235) of individuals in the high-risk category had high levels of TC, which was 3.25 times that in the low-risk group (8.98%, 118/1314). In the high-risk group, 36.03% (445/1235) of individuals had high LDL levels, which decreased to 23.06% (303/1314) in the low-risk group. Conversely, the proportion of individuals with high levels of HDL decreased from 6.16% (81/1314) in the low-risk group to 3.16% (39/1235) in the high-risk group.

## Discussion

HI is a prevalent sensorineural disorder and a growing public health issue of global concern. In this study, 57.3% were diagnosed with HFHI. It is suggested that half of all cases of HI can be prevented through public health measures [10, 37]. For instance, the World Health Organization (WHO) estimates that over 19% of childhood HI cases can be avoided by immunization against rubella and meningitis alone. In addition, the development and implementation of community-based hearing conservation programs aimed at changing listening behaviours and noise control in entertainment venues are also effective ways to intervene [38]. The development of a community-based risk screening tool for HI could be highly important and could be considered a first step in developing primary screening and prevention strategies for hearing health care in communities. In the current study, we adopted 7 ML algorithms to develop HFHI risk screening models by using questionnaire-based features and haematological test outcomes. The AUC values of the 6 models in the model validation stage were > 0.80, indicating that the ML model can be used to accurately identify HFHI individuals among ordinary residents.

When comparing the performance of the built models on the validation set, we found that the LASSO-based regression model achieved the best performance among all the algorithms tested, attaining an AUC of 0.868 (95% CI: 0.847–0.889) and reaching precision, specificity and F-score values all greater than 80% on the validation set. LASSO is a regression-based methodology allowing for simultaneous involvement of a large number of covariates in the model. More importantly, it penalizes the absolute value of a regression coefficient; thus, it is capable of regulating the impact a coefficient may have on the overall regression. The greater the penalization is, the greater the shrinkage of coefficients, with some reaching 0, thus automatically removing unnecessary/uninfluential covariates, making the model lean and resulting in a high discrimination rate [39]. The LASSO-based screening model is a classifier that can accumulate relative risks of meaningful variables and maximize its predictive power. This fundamental characteristic might explain the optimal discriminative ability of the LASSO model in our study [33].

Routine blood tests in primary community health care institutions are generally performed regularly at a frequency of at least once a year; on this basis, making full use of these indicators may promote primary screening and early warning of common diseases in community settings. In our study, the developed model revealed the value of haematological test data in screening HFHI, including metabolic parameters (e.g., LDL, HDL and TG), inflammatory parameters (e.g., NE and MPV) and other factors (e.g., AST). Among them, 4 indicators that had a significant impact on the screening model of the HFHI were TG, RDW, LY (%), and NE. Our research showed that residents with low TG levels and high TC levels are at greater risk of HFHI. Similarly, related studies have shown that TG, TC, and the LDL/HDL ratio are strongly associated with the prognosis of HI. When TC ranges from 5.2 to 6.2 mmol/L and TG ranges from 1.7 to 2.3 mmol/L, hypercholesterolemia increases the whole blood viscosity of the inner ear microvessels, which leads to haemorheological alterations and incomplete ischaemia [40]. A reasonable explanation is that abnormal blood lipid levels may lead to changes in whole blood viscosity, which may result in impaired microcirculation and ultimately lead to inadequate blood supply to target organs, resulting in sudden sensorineural HI [41]. In addition, our model revealed that high RDW is a risk factor for HFHI. Consistent with prior research, it has been shown that inflammatory markers are associated with HI in patients with inflammatory diseases, that there is a positive correlation between the RDW and mean hearing threshold, and that capturing inflammatory status is valuable for screening the risk of HI in residents with underlying chronic inflammation [42]. Furthermore, several social determinant features were recognized by our developed screening model as indicators of HFHI risk, including old age, male sex, low education level, and low income, which were all reported as risk factors for HI in previous studies [43]. Lifestyle factors such as smoking, alcohol drinking, a high volume when using electronic products and a high level of life pressure were also identified as risk factors for HFHI in our study. Previous research has shown that smokers are at a greater risk of HI and that the risk of smoking-related HI may decrease over time after quitting smoking [44]. There are many studies on the relationship between alcohol consumption and HI [45]. Some studies have found that moderate alcohol consumption has a protective effect on HI, which is similar to the cardiac protective effect of alcohol [46], whereas in contrast, our study found that alcohol consumption is a risk factor for HI. In addition, a cohort study has shown that individual music player users listening to high volumes increase the progression of their HI [47]. Therefore, timely intervention after early identification of risk factors in our model may have research significance for the occurrence of HFHI in residents.

The nomogram has been previously used as a predictive method in HI screening for occupational noise workers and achieved good screening results [48]. Therefore, to strengthen the operability and generalizability of our screening model for community physicians and residents, we also created an HFHI nomogram based on the derived LASSO-based HFHI risk model. Our nomogram achieved good calibration and discriminative ability in the validation cohort. This case study also revealed that this HFHI risk screening nomogram could be an effective tool for assessing HFHI risk in the general residential population and was especially valuable for screening potential HFHI patients in young or middle-aged adults. It is hoped that this nomogram could be further applied by primary care physicians as a routine tool at community health centres in the future to facilitate primary screening for HI and to help recognize a patient's modifiable risk factors for individualized intervention and treatment plans, ultimately driving current primary care towards personalized health care.

To our knowledge, this is the first study to combine residents' routine physical examination indicators and demographic and lifestyle risk factors from community residents to establish a risk assessment model for residents' HFHI based on an ML algorithm. Previous screening tools for HI have mostly targeted the elderly population, but they have primarily assessed the negative impact of HI on emotional and social problems and have not identified risk factors [49]. Model-based screening for HI achieved a better prediction effect, with AUC values ranging from 0.713–0.776, but it was mainly designed for noise-induced workers. Its risk factors are mainly industry type, noise exposure duration and median kurtosis [19, 50], which are quite different from those of ordinary residents. One study used demographics, clinical factors, and self-reported hearing status to predict whether speech frequency HI achieved good performance, but intervention after speech frequency HI was usually irreversible. The value of early prevention and intervention for HI is limited [21].

### Implications of the study

Our model has several potential implications and contributions. First, it encompassed a comprehensive array of variables, including basic personal information, current disease history, behavioural and environmental factors, and self-perceived hearing cognition. Additionally, it integrates haematological indicators at the biochemical level, thereby forming a screening model that systematically considers potential risk predictors across multiple dimensions, which can provide research ideas for the exploration of biomarkers related to HI. Second, we used and compared various ML algorithms to construct the model, and successfully proved the feasibility of using ML algorithms for developing HFHI screening models in the general population. More importantly, our screening tool is low-cost and widely accessible, making it suitable for deployment in grassroots community health service centers in China. In this context, community doctors can integrate inquiries about the HI risk factors into routine medical check-ups, leveraging our risk screening model to assess residents’ HI risk levels. As a result, the tool enables accurate identification of high-risk populations, making the initial stride towards early HI screening and prevention for community residents. Moreover, the model can help identify modifiable and intervenable risk factors among individuals at high risk of HI, thereby facilitating the delivery of precise interventions and targeted health guidance. Meanwhile, this model can also serve as an epidemiological tool to summarize shared and common HI risk factors at the population level. This application allows for the design of evidence-based prevention strategies and policy frameworks in public health, contributing significantly to proactive healthcare management and community well-being.

### Limitations

The limitations of this study need to be noted. First, the use of a cross-sectional design restricts the establishment of a direct causal relationship between identified predictors and the incidence of HFHI. Instead, this study provides suggestions from an aetiological perspective. Subsequent prospective studies are necessary to substantiate whether such associations are indicative of causal relationships. Second, our data were exclusively collected from seven health centres within a single province of China, potentially limiting the generalizability of the model to other regions in China with distinct socioeconomic and behavioural factors. In addition, the measurement of haematological data from different regions was conducted in the clinical laboratories of the investigated health care centres separately, which may introduce the potential for detection and measurement bias in this study.

## Conclusion

In this study, we developed and validated an HFHI risk screening model based on seven ML algorithms that included demographic information, symptoms and medical history, behavioural factors, environmental exposure, hearing cognition variables, and haematology test variables. Finally, the LASSO-based screening model achieved the best accuracy, and we further transformed it into a screenable nomogram that can be directly used in the community environment combined with electronic case information to conduct risk screening of the population. The model can classify community residents into different HFHI risk categories (high-, medium- and low-risk). The risk factors for each HI high-risk resident can be identified so that HI prevention or intervention can be personalized. Following this study, we intend to use the screening model to develop an early warning plug-in tailored for electronic medical record systems, with a specific focus on its implementation within primary care settings. This initiative aims to facilitate the ongoing collection of the latest data, allowing for a comprehensive external validation of the screening model on a larger scale, and eventually ascertain the viability of the instrument as a nationally applicable tool for future use.

### Supplementary Information

**Supplementary Material 1.**

**Supplementary Material 2. **

**Supplementary Material 3. **

**Supplementary Material 4. **

**Supplementary Material 5. **

## Data Availability

The datasets generated and/or analysed during the current study are not publicly available due to the owners considering further publications, but are available from the corresponding author on reasonable request.

## Abbreviations

ALB: Albumin
ALT: Alanine aminotransferase
AST: Aspartate aminotransferase
AUC: Area under curve
BA: Basophilic
BASO: Basophilic
BPC: Blood platelet count
BUN: Blood urea nitrogen
CI: Confidence interval
CR: Creatinine
dB: Decibel
DBIL: Direct bilirubin
EO: Eosinophil
FN: False negatives
FP: False positives
HCT: Hematocrit
HDL: High-density lipoprotein
HFHI: High-frequency hearing impairment
HGB: Hemoglobin
HI: Hearing impairment
IBIL: Indirect bilirubin
kHz: Kilohertz
KMO: Kaiser-Meyer-Olkin
KNN: K-nearest neighbors
LASSO: Least absolute shrinkage and selection operator
LDL: Low-density lipoprotein
LY: Lymphocyte
MCHC: Mean corpuscular hemoglobin concentration
MCH: Mean corpuscular hemoglobin
MCV: Mean corpuscular volume
ML: Machine learning
MO: Monocyte
MO: Monocyte
MPV: Mean platelet volume
NB: Naive Bayes
NE: Neutrophil
PDW: Platelet distribution width
PPV: Positive predictive value
RBC: Red blood cell
RDW: Red cell distribution width
RF: Random forest
ROC: Receiver operating characteristic
SVM: Support vector machine
TBIL: Total bilirubin
TC: Total cholesterol
TG: Triglyceride
TN: True negatives
TP: True positives
WBC: White blood cell
WHO: World Health Organization
XGBoost: eXtreme Gradient Boosting
