# Supplementary material for: A machine learning screening model for identifying the risk of high-frequency hearing impairment in a general population
Source: BMC Public Health. 2024 Apr 25;24:1160. doi: 10.1186/s12889-024-18636-1 (PMC11044481; doi:10.1186/s12889-024-18636-1)
Supplement: Supplementary file 1 — Supplementary Material 1. [file 12889_2024_18636_MOESM1_ESM.docx]

**Additional file 1.** The Questionnaire of this study.

**Hearing status survey form of residents in Zhejiang Province**

Dear Residents,

Hello! We are the Zhejiang Provincial Hearing Loss Investigation Research Team. Hearing loss has become a significant public health issue in our country, and the prevalence of hearing disabilities in Zhejiang Province exceeds the national average, making it even more deserving of attention. To comprehensively understand the hearing status and potential risk factors among Zhejiang residents, we have devised the following questionnaire. Your truthful responses are invaluable, and there are no right or wrong answers. Your privacy will be fully protected, and the data obtained will only be used for scientific research. If you have any questions about this study, please feel free to contact us. We sincerely appreciate your participation!

**Instructions for completion:** This form consists of sections of Demographics information, Symptom history, Disease history, Behavioral factors, Environmental Exposure, and Hearing cognitive conditions. Please fill in the data on the corresponding horizontal lines or check "√" on the options. Except for questions marked as "Multiple options", all are single-choice questions.

**1. Demographics information**

(1) Name: _________ (2) Age: years old

(3) Gender: □Male □Female

(4) Marital status: □Unmarried □Married □Divorced □Widowed

(5) Education level: □Primary schools and below

□Junior high school

□Senior high school or vocational school

□College or undergraduate

□Graduate or above

1. Personal average monthly income (RMB)：

□≤2000 □2001~ □4001~ □6001~ □≥8001

1. Is there a history of hereditary diseases in your family? □No □Yes__________

**2. Symptom histories**

(8) How would you rate your own hearing status?

□Good □Slight loss □Moderate loss □Severe loss □Extreme loss

1. Have you experienced tinnitus in the past year?

□No □Monthly occurrences □Weekly occurrences □Daily occurrences

1. Have you experienced ear pain in the past year?

□No □Monthly occurrences □Weekly occurrences □Daily occurrences

**3. Disease histories**

(11) Have you ever had any of the following diseases? **(Multiple options)**

□Hypertension □Diabetes □Cerebral haemorrhage □Arteriosclerosis

□Cerebral infection □Anemia □Migraine □Coronary heart disease □Otitis media □Chronic kidney disease □Tumors

**4. Behavioral factors**

(12) Do you smoke?

□Never □Former □Current

1. Are you regularly exposed to secondhand smoke in your daily life?

□No □1-2 days/week  □3-5 days/week □≥ 5 days/week

1. Do you drink alcohol?

□Never □Former □Current

1. How many hours do you usually sleep per night?

□<4 hours □ 4-6 hours □ 6-8 hours □ ≥ 8 hours

1. What is the volume level when you use headphones/earphones for phone calls, music, or watching videos?

□＜40% □40%-80% □≥80%

1. How much fruit and vegetables do you consume daily?

□＜500g □≥500g

1. How often do you engage in physical exercise/activities (such as running, playing sports, hiking)?

□Rarely □1-3 times/month □1-2 times/week

□3-4 times/week □>4 times/week

**5. Environmental Exposure**

1. Does your workplace environment suffer from noise pollution?

□Never or rarely □At least once a week □At least once a day

1. Is your residential environment affected by noise pollution?

□Never or rarely □At least once a week □At least once a day

1. How would you rate your work-related stress?

□Very high □High □Moderate □Relatively low □Very low

1. How would you rate your life-related stress?

□Very high □High □Moderate □Relatively low □Very low

**6. Hearing cognitive situation**

1. Have you ever paid attention to your hearing condition?

□Don't care □Not much attention □Average □Attention □Very concerned

1. Have you paid attention to knowledge about hearing protection?

□Don't care □Not much attention □Average □Attention □Very concerned

1. Do you think it is necessary to undergo regular hearing tests?

□Not necessary □Unsure □Needed □Necessary □Very necessary

1. Do you know how to protect your hearing?

□Don't know or know very little □Understand □Know

□Necessary □Very necessary

**Thank you for your cooperation!**

Surveyor: Survey date:____________________

Auditor: Audit date:____________________
