# Supplementary material for: A machine learning screening model for identifying the risk of high-frequency hearing impairment in a general population
Source: BMC Public Health. 2024 Apr 25;24:1160. doi: 10.1186/s12889-024-18636-1 (PMC11044481; doi:10.1186/s12889-024-18636-1)
Supplement: Supplementary file 2 — Supplementary Material 2. [file 12889_2024_18636_MOESM2_ESM.docx]

**Additional file 2.** The range of reference values for hematological variables in this study.

| **Hematological variables** | **Unit** | **References** | | | | | | | |
| --- | --- | --- | --- | --- | --- | --- | --- | --- | --- |
|  |  | **Anji** | **Baiyang** | **Jiangshan** | **Jiaxing** | **Lishui** | **Sijiqing** | **Tonglu** |  |
| EO | % | 0.4-8.0 | 0.0-6.0 | 0.4-8.0 | 0.4-8.0 | 0.4-8.0 | 0.5-5.0 | 0.5-5.0 |  |
| BA | 10^9/L | 0.0-0.06 | 0.0-0.2 | 0.0-0.06 | 0.0-0.1 | 0.0-0.06 | 0.0-0.05 | 0.0-0.2 |  |
| EO | 10^9/L | 0.02-0.52 | 0.02-0.52 | 0.02-0.52 | 0.02-0.52 | 0.02-0.52 | 0.05-0.30 | 0.03-0.5 |  |
| HGB | g/L | 115.0-150.0 | 110.0-160.0 | 130.0-175.0 | 130.0-175.0 | 130.0-175.0 | 120.0-170.0 | 120.0-180.0 |  |
| LY | % | 20.0-50.0 | 20.0-40.0 | 20.0-50.0 | 20.0-50.0 | 20.0-50.0 | 20.0-40.0 | 20.0-40.0 |  |
| MCHC | g/L | 316.0-354.0 | 320.0-360.0 | 316.0-354.0 | 316.0-354.0 | 316.0-354.0 | 300.0-380.0 | 316.0-360.0 |  |
| MO | 10^9/L | 0.1-0.6 | 0.1-0.9 | 0.1-0.6 | 0.1-0.6 | 0.1-0.6 | 0.12-1.00 | 0.1-1.0 |  |
| MPV | f L | 6.5-13.0 | 5.0-11.0 | 7.4-12.0 | 6.5-13.0 | 9.0-13.0 | 4.5-11.5 | 8.0-13.0 |  |
| NE | % | 40.0-75.0 | 50.0-70.0 | 40.0-75.0 | 40.0-75.0 | 40.0-75.0 | 50.0-70.0 | 43.0-72.0 |  |
| BPC | 10^9/L | 125.0-350.0 | 100.0-300.0 | 125.0-350.0 | 125.0-350.0 | 125.0-350.0 | 100.0-300.0 | 85.0-300.0 |  |
| RDW | % | 11.0-16.0 | 11.5-14.5 | 11.6-13.7 | 0.106-0.15 | 11.0-16.0 | 11.5-15.0 | 10.0-15.0 |  |
| BASO | % | 0.0-1.0 | 0.0-2.0 | 0.0-1.0 | 0.0-2.0 | 0.0-1.0 | 0.0-1.0 | 0.0-1.2 |  |
| HCT | % | 35.0-45.0 | 37.0-50.0 | 40-50 | 40.0-50.0 | 40.0-50.0 | 38.0-50.2 | 37.0-50.0 |  |
| LY | 10^9/L | 1.1-3.2 | 0.8-4.0 | 1.1-3.2 | 1.1-3.2 | 1.1-3.2 | 1.0-3.3 | 0.8-4.0 |  |
| MCH | pg | 27.0-34.0 | 27.0-34.0 | 27.0-34.0 | 27.0-34.0 | 27.0-34.0 | 28.0-34.0 | 27.0-33.0 |  |
| MCV | fL | 82.0-100.0 | 82.0-95.0 | 82.0-100.0 | 82.0-100.0 | 82.0-100.0 | 80.0-100.0 | 86.0-100.0 |  |
| MO | % | 3.0-10.0 | 3.0-9.0 | 3.0-10.0 | 3.0-10.0 | 3.0-10.0 | 3.0-8.0 | 3.0-10.0 |  |
| NE | 10^9/L | 1.8-6.3 | 2.0-7.0 | 1.8-6.3 | 1.8-6.3 | 1.8-6.3 | 1.8-6.4 | 1.8-7.2 |  |
| PDW | % | 9.8-16.2 | 15.0-20.0 | 12.0-18.0 | 9.0-17.0 | 9.0-17.0 | 10.3-20.0 | 9.0-18.0 |  |
| RBC | 10^12/L | 3.8-5.1 | 3.5-5.5 | 4.3-5.8 | 4.3-5.8 | 4.3-5.8 | 4.09-5.74 | 4.00-5.50 |  |
| WBC | 10^9/L | 3.5-9.5 | 4.0-10.0 | 3.5-9.5 | 3.5-9.5 | 3.5-9.5 | 4.0-10.0 | 4.0-10.0 |  |
| TG | m mol/L | 0.48-2.26 | 0.45-2.26 | 0.56-1.70 | 0.48-1.88 | 0.34-1.69 | 0.4-1.8 | 0.57-1.70 |  |
| ALT | U/L | 1.0-50.0 | 0.0-50.0 | 9.0-50.0 | 9.0-50.0 | 9.0-50.0 | 9.0-50.0 | 0.0-50.0 |  |
| IBIL | μ mol/L | 0.0-17.0 | 0.0-18.0 | 3.0-19.0 | 0.0-13.7 | 1.3-14.2 | 2.0-15.0 | 0.1-16.5 |  |
| DBIL | μ mol/L | 0.0-0.7 | 0.0-10.0 | 1.7-8.0 | 0.0-10.3 | 0.0-7.8 | 0.0-7.0 | 0.0-6.8 |  |
| ALB | g/L | 35.0-55.0 | 35.0-55.0 | 40.0-55.0 | 40.0-55.0 | 40.0-55.0 | 40.0-55.0 | 35.0-55.0 |  |
| TBIL | μ mol/L | 5.1-22.0 | 0.0-28.0 | 3.4-25.0 | 3.4-20.5 | 3.0-22.0 | 3.0-20.0 | 3.0-21.0 |  |
| BUN | m mol/L | 1.79-7.14 | 3.5-7.2 | 1.7-7.1 | 3.1-8.0 | 2.9-8.2 | 3.1-8.0 | 1.43-7.14 |  |
| AST | U/L | 1.0-40.0 | 0.0-50.0 | 15.0-40.0 | 15.0-40.0 | 15.0-40.0 | 15.0-40.0 | 0.0-50.0 |  |
| TC | m mol/L | 3.35-6.45 | 3.23-5.17 | 3.1-5.95 | 3.35-6.45 | 2.84-5.17 | 3.0-6.0 | 2.33-5.17 |  |
| LDL | m mol/L | 0.9-4.11 | 0.0-3.4 | 1.07-3.10 | 2.06-3.1 | 0.0-3.12 | 0.0-3.12 | 0.0-3.12 |  |
| HDL | m mol/L | 0.91-2.27 | 0.91-2.0 | 1.01-1.98 | 0.77-2.25 | >1.04 | 0.90-1.66 | 1.04-2.50 |  |
| CR | μ mol/L | 45.0-115.0 | 44.0-133.0 | 40.0-115.0 | 44.0-120.0 | 59.0-104.0 | 57.0-97.0 | 44.0-97.0 |  |
