# Supplementary material for: A machine learning screening model for identifying the risk of high-frequency hearing impairment in a general population
Source: BMC Public Health. 2024 Apr 25;24:1160. doi: 10.1186/s12889-024-18636-1 (PMC11044481; doi:10.1186/s12889-024-18636-1)
Supplement: Supplementary file 3 — Supplementary Material 3. [file 12889_2024_18636_MOESM3_ESM.docx]

**Additional file 3.** List of characteristics collected from various sources and categories, and their univariates comparison results between HFHI Cases and Controls.

| **Characteristic** | **Study** | **Normal** | **HFHI** | ***P*-value** |
| --- | --- | --- | --- | --- |
|  | **cohort (%)** | **n (%)** | **n (%)** |  |
|  | **(N=3371)** | **(N=1441)** | **(N=1930)** |  |
| **1. Demographics** |  |  |  |  |
| **Age（years）** |  |  |  |  |
| 18-25 | 119 (3.5) | 108 (7.5) | 11 (0.6) | <0.001 |
| 26-35 | 566 (16.8) | 455 (31.6) | 111 (5.8) |  |
| 36-45 | 634 (18.8) | 399 (27.7) | 235 (12.2) |  |
| 46-55 | 725 (21.5) | 302 (21.0) | 423 (21.9) |  |
| 56-65 | 745 (22.1) | 133 (9.2) | 612 (31.7) |  |
| 66-75 | 416 (12.3) | 35 (2.4) | 381 (19.7) |  |
| ≥76 | 166 (5.0) | 9 (0.6) | 157 (8.1) |  |
| **Gender** |  |  |  |  |
| Male | 1730 (51.3) | 660 (45.8) | 1070 (55.4) | <0.001 |
| Female | 1641 (48.7) | 781 (54.2) | 860 (44.6) |  |
| **Marital status** |  |  |  |  |
| Unmarried | 215 (6.4) | 176 (12.2) | 39 (2.0) | <0.001 |
| Married | 3052 (90.5) | 1238 (85.9) | 1814 (94.0) |  |
| Divorce | 57 (1.7) | 21 (1.5) | 36 (1.9) |  |
| Widowed | 47 (1.4) | 6 (0.4) | 41 (2.1) |  |
| **Education level** |  |  |  |  |
| Primary schools and below | 488 (14.5) | 57 (4.0) | 431 (22.3) | <0.001 |
| Junior high school | 663 (19.7) | 179 (12.4) | 484 (25.1) |  |
| Senior high school or vocational school | 830 (24.6) | 338 (23.5) | 492 (25.5) |  |
| College or Undergraduate | 1356 (40.2) | 845 (58.6) | 511 (26.5) |  |
| Graduate and above | 34 (1.0) | 22 (1.5) | 12 (0.6) |  |
| **Personal average monthly income (RMB)** |  |  |  |  |
| ≤2000 | 547 (16.2) | 85 (6.0) | 462 (23.9) | <0.001 |
| 2001~ | 826 (24.5) | 342 (23.7) | 484 (25.1) |  |
| 4001~ | 1262 (37.4) | 639 (44.3) | 623 (32.3) |  |
| 6001~ | 561 (16.6) | 285 (19.8) | 276 (14.3) |  |
| ≥8001 | 175 (5.3) | 90 (6.2) | 85 (4.4) |  |
| **Familial disease** |  |  |  |  |
| No | 3109 (92.2) | 1380 (95.8) | 1729 (89.6) | <0.001 |
| Yes | 262 (7.8) | 61 (4.2) | 201 (10.4) |  |
| **2. Symptom histories** |  |  |  |  |
| **Self-perceived hearing status** |  |  |  |  |
| Good | 2251 (66.8) | 1255 (87.1) | 996 (51.6) | <0.001 |
| Slight loss | 821 (24.4) | 166 (11.5) | 655 (33.9) |  |
| Moderate loss | 239 (7.1) | 19 (1.3) | 220 (11.5) |  |
| Severe loss | 49 (1.5) | 0 (0.0) | 49 (2.5) |  |
| Extreme loss | 11 (0.2) | 1 (0.1) | 10 (0.5) |  |
| **Tinnitus history in the past year** |  |  |  |  |
| No | 2639 (78.3) | 1235 (85.8) | 1404 (72.7) | <0.001 |
| Monthly | 472 (14.0) | 166 (11.5) | 306 (15.9) |  |
| Weekly | 158 (4.7) | 25 (1.7) | 133 (6.9) |  |
| Daily | 102 (3.0) | 15 (1.0) | 87 (4.5) |  |
| **Ear pain history in the past year** |  |  |  |  |
| No | 3195 (94.8) | 1406 (97.6) | 1789 (92.7) | <0.001 |
| Monthly | 125 (3.7) | 30 (2.1) | 95 (4.9) |  |
| Weekly | 44 (1.3) | 3 (0.2) | 41 (2.1) |  |
| Daily | 7 (0.2) | 2 (0.1) | 5 (0.3) |  |
| **3. Disease histories** |  |  |  |  |
| **Hypertension** |  |  |  |  |
| No | 2637 (78.2) | 1346 (93.4) | 1291 (66.9) | <0.001 |
| Yes | 734 (21.8) | 95 (6.6) | 639 (33.1) |  |
| **Diabetes** |  |  |  |  |
| No | 3225 (95.7) | 1430 (99.2) | 1795 (93.0) | <0.001 |
| Yes | 146 (4.3) | 11 (0.8) | 135 (7.0) |  |
| **Cerebral hemorrhage** |  |  |  |  |
| No | 3365 (99.8) | 1440 (99.9) | 1925 (99.7) | 0.379 |
| Yes | 6 (0.2) | 1 (0.1) | 5 (0.3) |  |
| **Arteriosclerosis** |  |  |  |  |
| No | 3360 (99.7) | 1441 (100.0) | 1919 (99.4) | 0.01 |
| Yes | 11 (0.3) | 0 (0.0) | 11 (0.6) |  |
| **Cerebral infection** |  |  |  |  |
| No | 3360 (99.7) | 1438 (99.8) | 1922 (99.6) | 0.463 |
| Yes | 11 (0.3) | 3 (0.2) | 8 (0.4) |  |
| **Anemia** |  |  |  |  |
| No | 3243 (96.2) | 1383 (96.0) | 1860 (96.4) | 0.612 |
| Yes | 128 (3.8) | 58 (4.0) | 70 (3.6) |  |
| **Migraine** |  |  |  |  |
| No | 3256 (96.6) | 1400 (97.2) | 1856 (96.2) | 0.142 |
| Yes | 115 (3.4) | 41 (2.8) | 74 (3.8) |  |
| **Coronary heart disease** |  |  |  |  |
| No | 3354 (99.5) | 1441 (100.0) | 1913 (99.1) | 0.001 |
| Yes | 17 (0.5) | 0 (0.0) | 17 (0.9) |  |
| **Otitis media** |  |  |  |  |
| No | 3293 (97.7) | 1429 (99.2) | 1864 (96.6) | <0.001 |
| Yes | 78 (2.3) | 12 (0.8) | 66 (3.4) |  |
| **Chronic kidney disease** |  |  |  |  |
| No | 3368 (99.9) | 1440 (99.9) | 1928 (99.9) | 1 |
| Yes | 3 (0.1) | 1 (0.1) | 2 (0.1) |  |
| **Tumors** |  |  |  |  |
| No | 3359 (99.6) | 1438 (99.8) | 1921 (99.5) | 0.341 |
| Yes | 12 (0.4) | 3 (0.2) | 9 (0.5) |  |
| **4. Behavioral factors** |  |  |  |  |
| **Smoking** |  |  |  |  |
| Never | 2472 (73.4) | 1197 (83.1) | 1275 (66.1) | <0.001 |
| Former | 203 (6.0) | 29 (2.0) | 174 (9.0) |  |
| Current | 696 (20.6) | 215 (14.9) | 481 (24.9) |  |
| **Secondhand smoking** |  |  |  |  |
| No | 1878 (55.7) | 801 (55.6) | 1077 (55.8) | <0.001 |
| 1-2 days/week | 693 (20.5) | 346 (24.0) | 347 (18.0) |  |
| 3-5 days/week | 370 (11.0) | 162 (11.2) | 208 (10.8) |  |
| ≥ 5 days/week | 430 (12.8) | 132 (9.2) | 298 (15.4) |  |
| **Alcohol drinking** |  |  |  |  |
| Never | 2741 (81.3) | 1266 (87.9) | 1475 (76.4) | <0.001 |
| Former | 61 (1.8) | 15 (1.0) | 46 (2.4) |  |
| Current | 569 (16.9) | 160 (11.1) | 409 (21.2) |  |
| **Hours of sleep** |  |  |  |  |
| ＜4 hours | 20 (0.6) | 4 (0.3) | 16 (0.8) | <0.001 |
| 4-6 hours | 203 (6.0) | 63 (4.3) | 140 (7.3) |  |
| 6-8 hours | 2257 (67.0) | 1023 (71.0) | 1234 (63.9) |  |
| ≥ 8 hours | 891 (26.4) | 351 (24.4) | 540 (28.0) |  |
| **Electronic volume** |  |  |  |  |
| ＜40% | 1057 (31.4) | 556 (38.6) | 501 (26.0) | <0.001 |
| 40%-80% | 1685 (50.0) | 745 (51.7) | 940 (48.7) |  |
| ≥80% | 629 (18.6) | 140 (9.7) | 489 (25.3) |  |
| **Daily fruit and vegetable intaking** |  |  |  |  |
| ＜500g | 1396 (41.4) | 493 (34.2) | 903 (46.8) | <0.001 |
| ≥500g | 1975 (58.6) | 948 (65.8) | 1027 (53.2) |  |
| **Exercise frequency** |  |  |  |  |
| Rarely | 1338 (39.7) | 448 (31.1) | 890 (46.1) | <0.001 |
| 1-3 times/month | 638 (18.9) | 326 (22.6) | 312 (16.2) |  |
| 1-2 times/week | 548 (16.3) | 286 (19.8) | 262 (13.5) |  |
| 3-4 times/week | 312 (9.2) | 187 (13.0) | 125 (6.5) |  |
| ＞ 4 times/week | 535 (15.9) | 194 (13.5) | 341 (17.7) |  |
| **5. Environmental Exposure** |  |  |  |  |
| **Workplace noise exposure** |  |  |  |  |
| Never or rarely | 2130 (63.2) | 944 (65.5) | 1186 (61.5) | <0.001 |
| At least once a week | 850 (25.2) | 368 (25.5) | 482 (25.0) |  |
| At least once a day | 391 (11.6) | 129 (9.0) | 262 (13.5) |  |
| **Living noise exposure** |  |  |  |  |
| Never or rarely | 2583 (76.6) | 1131 (78.5) | 1452 (75.2) | 0.053 |
| At least once a week | 517 (15.3) | 210 (14.6) | 307 (15.9) |  |
| At least once a day | 271 (8.1) | 100 (6.9) | 171 (8.9) |  |
| **Work stress** |  |  |  |  |
| Very high | 80 (2.4) | 44 (3.1) | 36 (1.9) | <0.001 |
| High | 785 (23.3) | 309 (21.4) | 476 (24.7) |  |
| Moderate | 1358 (40.3) | 677 (47.0) | 681 (35.3) |  |
| Relatively low | 765 (22.7) | 344 (23.9) | 421 (21.7) |  |
| Very low | 383 (11.3) | 67 (4.6) | 316 (16.4) |  |
| **Life stress** |  |  |  |  |
| Very high | 78 (2.3) | 36 (2.5) | 42 (2.2) | <0.001 |
| High | 533 (15.8) | 185 (12.8) | 348 (18.0) |  |
| Moderate | 1464 (43.4) | 728 (50.5) | 736 (38.1) |  |
| Relatively low | 885 (26.3) | 397 (27.6) | 488 (25.3) |  |
| Very low | 411 (12.2) | 95 (6.6) | 316 (16.4) |  |
| **6. Hearing cognitive situation** |  |  |  |  |
| **Pay attention to your hearing** |  |  |  |  |
| Don't care | 567 (16.8) | 272 (18.9) | 295 (15.3) | <0.001 |
| Not much attention | 982 (29.1) | 407 (28.2) | 575 (29.8) |  |
| Average | 1075 (31.9) | 484 (33.6) | 591 (30.6) |  |
| Attention | 601 (17.8) | 258 (17.9) | 343 (17.8) |  |
| Very concerned | 146 (4.4) | 20 (1.4) | 126 (6.5) |  |
| **Pay attention to hearing protection** |  |  |  |  |
| Don't care | 627 (18.6) | 277 (19.2) | 350 (18.1) | <0.001 |
| Not much attention | 892 (26.5) | 374 (26.0) | 518 (26.8) |  |
| Average | 1163 (34.5) | 538 (37.3) | 625 (32.4) |  |
| Attention | 556 (16.5) | 232 (16.1) | 324 (16.8) |  |
| Very concerned | 133 (3.9) | 20 (1.4) | 113 (5.9) |  |
| **Regular hearing check** |  |  |  |  |
| Not necessary | 467 (13.9) | 206 (14.3) | 261 (13.5) | 0.007 |
| Unsure | 1294 (38.4) | 512 (35.5) | 782 (40.5) |  |
| Needed | 1352 (40.1) | 621 (43.1) | 731 (37.9) |  |
| Necessary | 204 (6.0) | 85 (5.9) | 119 (6.2) |  |
| Very necessary | 54 (1.6) | 17 (1.2) | 37 (1.9) |  |
| **Hearing protection skills** |  |  |  |  |
| Don't know or know very little | 1794 (53.2) | 747 (51.8) | 1047 (54.2) | <0.001 |
| Understand | 1023 (30.3) | 472 (32.8) | 551 (28.5) |  |
| know | 199 (5.9) | 104 (7.2) | 95 (4.9) |  |
| Necessary | 181 (5.4) | 86 (6.0) | 95 (4.9) |  |
| Very necessary | 174 (5.2) | 32 (2.2) | 142 (7.5) |  |
| **7. Blood Routine Indices** |  |  |  |  |
| **EO (%)** |  |  |  |  |
| Low | 57 (1.7) | 26 (1.8) | 31 (1.6) | <0.001 |
| Normal | 3192 (94.7) | 1385 (96.1) | 1807 (93.6) |  |
| High | 122 (3.6) | 30 (2.1) | 92 (4.8) |  |
| **BA** |  |  |  |  |
| Normal | 2747 (81.5) | 1126 (78.1) | 1621 (84.0) | <0.001 |
| High | 624 (18.5) | 315 (21.9) | 309 (16.0) |  |
| **EO** |  |  |  |  |
| Low | 116 (3.4) | 34 (2.3) | 82 (4.2) | <0.001 |
| Normal | 2609 (77.4) | 1095 (76.0) | 1514 (78.5) |  |
| High | 646 (19.2) | 312 (21.7) | 334 (17.3) |  |
| **HGB** |  |  |  |  |
| Low | 931 (27.6) | 480 (33.3) | 451 (23.4) | <0.001 |
| Normal | 2398 (71.2) | 943 (65.4) | 1455 (75.4) |  |
| High | 42 (1.2) | 18 (1.3) | 24 (1.2) |  |
| **LY (%)** |  |  |  |  |
| Low | 664 (19.7) | 325 (22.6) | 339 (17.6) | <0.001 |
| Normal | 2373 (70.4) | 1015 (70.4) | 1358 (70.4) |  |
| High | 334 (9.9) | 101 (7.0) | 233 (12.0) |  |
| **MCHC** |  |  |  |  |
| Low | 816 (24.2) | 395 (27.4) | 421 (21.8) | 0.001 |
| Normal | 2536 (75.2) | 1038 (72.0) | 1498 (77.6) |  |
| High | 19 (0.6) | 8 (0.6) | 11 (0.6) |  |
| **MO** |  |  |  |  |
| Low | 3 (0.1) | 2 (0.1) | 1 (0.1) | 0.002 |
| Normal | 2751 (81.6) | 1137 (78.9) | 1614 (83.6) |  |
| High | 617 (18.3) | 302 (21.0) | 315 (16.3) |  |
| **MPV** |  |  |  |  |
| Low | 594 (17.6) | 291 (20.2) | 303 (15.7) | 0.001 |
| Normal | 2414 (71.6) | 1015 (70.4) | 1399 (72.5) |  |
| High | 363 (10.8) | 135 (9.4) | 228 (11.8) |  |
| **NE (%)** |  |  |  |  |
| Low | 734 (21.8) | 341 (23.7) | 393 (20.4) | 0.051 |
| Normal | 2564 (76.0) | 1073 (74.5) | 1491 (77.2) |  |
| High | 73 (2.2) | 27 (1.8) | 46 (2.4) |  |
| **BPC** |  |  |  |  |
| Low | 646 (19.2) | 310 (21.5) | 336 (17.4) | <0.001 |
| Normal | 2609 (77.4) | 1069 (74.2) | 1540 (79.8) |  |
| High | 116 (3.4) | 62 (4.3) | 54 (2.8) |  |
| **RDW** |  |  |  |  |
| Low | 777 (23.0) | 419 (29.0) | 358 (18.5) | <0.001 |
| Normal | 2396 (71.1) | 962 (66.8) | 1434 (74.3) |  |
| High | 198 (5.9) | 60 (4.2) | 138 (7.2) |  |
| **BASO (%)** |  |  |  |  |
| Normal | 3308 (98.1) | 1415 (98.2) | 1893 (98.1) | 0.912 |
| High | 63 (1.9) | 26 (1.8) | 37 (1.9) |  |
| **HCT** |  |  |  |  |
| Low | 991 (29.4) | 504 (35.0) | 487 (25.2) | <0.001 |
| Normal | 2271 (67.4) | 885 (61.4) | 1386 (71.8) |  |
| High | 109 (3.2) | 52 (3.6) | 57 (3.0) |  |
| **LY** |  |  |  |  |
| Low | 605 (17.9) | 299 (20.7) | 306 (15.9) | <0.001 |
| Normal | 2650 (78.6) | 1083 (75.2) | 1567 (81.1) |  |
| High | 116 (3.5) | 59 (4.1) | 57 (3.0) |  |
| **MCH** |  |  |  |  |
| Low | 724 (21.5) | 369 (25.6) | 355 (18.3) | <0.001 |
| Normal | 2578 (76.5) | 1060 (73.6) | 1518 (78.7) |  |
| High | 69 (2.0) | 12 (0.8) | 57 (3.0) |  |
| **MCV** |  |  |  |  |
| Low | 705 (20.9) | 352 (24.4) | 353 (18.3) | <0.001 |
| Normal | 2544 (75.5) | 1073 (74.5) | 1471 (76.2) |  |
| High | 122 (3.6) | 16 (1.1) | 106 (5.5) |  |
| **MO (%)** |  |  |  |  |
| Low | 608 (18.0) | 312 (21.7) | 296 (15.3) | <0.001 |
| Normal | 2629 (78.0) | 1093 (75.9) | 1536 (79.6) |  |
| High | 134 (4.0) | 36 (2.4) | 98 (5.1) |  |
| **NE** |  |  |  |  |
| Low | 623 (18.5) | 302 (21.0) | 321 (16.6) | 0.001 |
| Normal | 2675 (79.4) | 1101 (76.4) | 1574 (81.6) |  |
| High | 73 (2.1) | 38 (2.6) | 35 (1.8) |  |
| **PDW** |  |  |  |  |
| Low | 1329 (39.4) | 693 (48.1) | 636 (33.0) | <0.001 |
| Normal | 1864 (55.3) | 684 (47.5) | 1180 (61.1) |  |
| High | 178 (5.3) | 64 (4.4) | 114 (5.9) |  |
| **RBC** |  |  |  |  |
| Low | 1097 (32.5) | 448 (31.1) | 649 (33.6) | 0.298 |
| Normal | 2207 (65.5) | 964 (66.9) | 1243 (64.4) |  |
| High | 67 (2.0) | 29 (2.0) | 38 (2.0) |  |
| **WBC** |  |  |  |  |
| Low | 641 (19.0) | 301 (20.9) | 340 (17.6) | <0.001 |
| Normal | 2653 (78.7) | 1094 (75.9) | 1559 (80.8) |  |
| High | 77 (2.3) | 46 (3.2) | 31 (1.6) |  |
| **8. Hepatic Function Indices** |  |  |  |  |
| **TG** |  |  |  |  |
| Low | 296 (8.8) | 173 (12.0) | 123 (6.4) | <0.001 |
| Normal | 2353 (69.8) | 981 (68.1) | 1372 (71.1) |  |
| High | 722 (21.4) | 287 (19.9) | 435 (22.5) |  |
| **ALT** |  |  |  |  |
| Low | 33 (1.0) | 21 (1.5) | 12 (0.6) | 0.046 |
| Normal | 3148 (93.4) | 1336 (92.7) | 1812 (93.9) |  |
| High | 190 (5.6) | 84 (5.8) | 106 (5.5) |  |
| **IBIL** |  |  |  |  |
| Low | 56 (1.6) | 37 (2.6) | 19 (1.0) | 0.001 |
| Normal | 3046 (90.4) | 1300 (90.2) | 1746 (90.5) |  |
| High | 269 (8.0) | 104 (7.2) | 165 (8.5) |  |
| **DBIL** |  |  |  |  |
| Low | 99 (3.0) | 50 (3.5) | 49 (2.5) | 0.006 |
| Normal | 3153 (93.5) | 1355 (94.0) | 1798 (93.2) |  |
| High | 119 (3.5) | 36 (2.5) | 83 (4.3) |  |
| **ALB** |  |  |  |  |
| Low | 619 (18.4) | 306 (21.2) | 313 (16.2) | 0.001 |
| Normal | 2742 (81.3) | 1131 (78.5) | 1611 (83.5) |  |
| High | 10 (0.3) | 4 (0.3) | 6 (0.3) |  |
| **TBIL** |  |  |  |  |
| Low | 563 (16.7) | 284 (19.7) | 279 (14.5) | <0.001 |
| Normal | 2552 (75.7) | 1065 (73.9) | 1487 (77.0) |  |
| High | 256 (7.6) | 92 (6.4) | 164 (8.5) |  |
| **BUN** |  |  |  |  |
| Low | 599 (17.8) | 302 (21.0) | 297 (15.4) | <0.001 |
| Normal | 2568 (76.2) | 1109 (77.0) | 1459 (75.6) |  |
| High | 204 (6.0) | 30 (2.0) | 174 (9.0) |  |
| **AST** |  |  |  |  |
| Low | 144 (4.3) | 103 (7.2) | 41 (2.1) | <0.001 |
| Normal | 3109 (92.2) | 1290 (89.5) | 1819 (94.3) |  |
| High | 118 (3.5) | 48 (3.3) | 70 (3.6) |  |
| **TC** |  |  |  |  |
| Low | 928 (27.6) | 471 (32.7) | 457 (23.7) | <0.001 |
| Normal | 1818 (53.9) | 803 (55.7) | 1015 (52.6) |  |
| High | 625 (18.5) | 167 (11.6) | 458 (23.7) |  |
| **LDL** |  |  |  |  |
| Low | 99 (2.9) | 54 (3.7) | 45 (2.3) | <0.001 |
| Normal | 2290 (67.9) | 1028 (71.4) | 1262 (65.4) |  |
| High | 982 (29.0) | 359 (24.9) | 623 (32.3) |  |
| **HDL** |  |  |  |  |
| Low | 124 (3.7) | 46 (3.2) | 78 (4.0) | 0.001 |
| Normal | 3102 (92.0) | 1313 (91.1) | 1789 (92.7) |  |
| High | 145 (4.3) | 82 (5.7) | 63 (3.3) |  |
| **CR** |  |  |  |  |
| Low | 715 (21.2) | 376 (26.1) | 339 (17.6) | <0.001 |
| Normal | 2586 (76.7) | 1053 (73.1) | 1533 (79.4) |  |
| High | 70 (2.1) | 12 (0.8) | 58 (3.0) |  |
