# Supplementary material for: A machine learning screening model for identifying the risk of high-frequency hearing impairment in a general population
Source: BMC Public Health. 2024 Apr 25;24:1160. doi: 10.1186/s12889-024-18636-1 (PMC11044481; doi:10.1186/s12889-024-18636-1)
Supplement: Supplementary file 4 — Supplementary Material 4. [file 12889_2024_18636_MOESM4_ESM.docx]

**Additional file 4.** Comparison of prediction results of cross-validation models

| **Model** | **Original[AUC(95%CI)]** | **cv.5 [AUC(95%CI]** | **cv.10 [AUC(95%CI)]** |
| --- | --- | --- | --- |
| LASSO | 0.868 (0.847-0.889) | 0.857 (0.845-0.870) | 0.857 (0.844-0.869) |
| XGBoost | 0.854 (0.833-0.876) | 0.823 (0.809-0.837) | 0.828 (0.814-0.842) |
| RF | 0.803 (0.780-0.827) | 0.794 (0.780-0.808) | 0.798 (0.784-0.812) |
| SVM | 0.805 (0.781-0.828) | 0.786 (0.772-0.800) | 0.792 (0.778-0.806) |
| KNN | 0.866 (0.845-0.887) | 0.857 (0.844-0.869) | 0.858 (0.845-0.870) |
| NB | 0.653 (0.634-0.673) | 0.656 (0.644-0.668) | 0.644 (0.632-0.656) |
| Boosting | 0.858 (0.837-0.880) | 0.843 (0.830-0.856) | 0.854 (0.841-0.867) |
