# Supplementary material for: A machine learning screening model for identifying the risk of high-frequency hearing impairment in a general population
Source: BMC Public Health. 2024 Apr 25;24:1160. doi: 10.1186/s12889-024-18636-1 (PMC11044481; doi:10.1186/s12889-024-18636-1)
Supplement: Supplementary file 5 — Supplementary Material 5. [file 12889_2024_18636_MOESM5_ESM.docx]

**Additional file 5.** The estimated coefficients in the LASSO regression.

| **Variable** | **LASSO regression** |
| --- | --- |
| Intercept | -3.516 |
| **Demographics** |  |
| Age (years) | 0.721 |
| Gender | -0.273 |
| Education level | -0.203 |
| Personal average monthly income (RMB) | -0.096 |
| Familial disease | 0.134 |
| **Symptom histories** |  |
| Self-perceived hearing status | 0.762 |
| Tinnitus history in the past year | 0.060 |
| **Diseases histories** |  |
| Hypertension | 0.540 |
| Diabetes | 0.232 |
| Arteriosclerosis | 0.126 |
| Coronary heart disease | 1.122 |
| Otitis media | 0.965 |
| **Behavioral factors** |  |
| Smoking | 0.103 |
| Alcohol drinking | 0.005 |
| Electronic volume | 0.154 |
| Daily fruit and vegetable intaking | 0.027 |
| Exercise frequency | -0.031 |
| **Environmental exposure** |  |
| Workplace noise exposure | 0.023 |
| Life stress | -0.149 |
| **Hearing cognitive situation** |  |
| Pay attention to your hearing | 0.034 |
| Pay attention to hearing protection | 0.108 |
| **Blood Routine Indices** |  |
| EO (%) | 0.035 |
| HGB | 0.169 |
| LY (%) | 0.257 |
| MPV | -0.118 |
| RDW | 0.368 |
| NE | -0.129 |
| PDW | 0.292 |
| **Hepatic Function Indices** |  |
| TG | -0.231 |
| IBIL | 0.165 |
| AST | 0.023 |
| TC | 0.114 |
| LDL | 0.044 |
| HDL | -0.109 |
